# Purging of inbreeding depression within the Irish Holstein-Friesian population

**DOI:** 10.1186/1297-9686-41-16

**Published:** 2009-01-21

**Authors:** Sinéad Mc Parland, Francis Kearney, Donagh P Berry

**Affiliations:** 1 Teagasc, Moorepark Dairy Production Research Centre, Fermoy, Co. Cork, Ireland; 2 Animal Genomics Laboratory, School of Agriculture, Food Science and Veterinary Medicine and Conway Institute of Biomolecular and Biomedical Research, College of Life Sciences, University College Dublin, Belfield, Dublin 4, Ireland; 3 Irish Cattle Breeding Federation, Bandon, Co. Cork, Ireland

## Abstract

The objective of this study was to investigate whether inbreeding depression in milk production or fertility performance has been partially purged due to selection within the Irish Holstein-Friesian population. Classical, ancestral (*i.e*., the inbreeding of an individual's ancestors according to two different formulae) and new inbreeding coefficients (*i.e*., part of the classical inbreeding coefficient that is not accounted for by ancestral inbreeding) were computed for all animals. The effect of each coefficient on 305-day milk, fat and protein yield as well as calving interval, age at first calving and survival to second lactation was investigated. Ancestral inbreeding accounting for all common ancestors in the pedigree had a positive effect on 305-day milk and protein yield, increasing yields by 4.85 kg and 0.12 kg, respectively. However, ancestral inbreeding accounting only for those common ancestors, which contribute to the classical inbreeding coefficient had a negative effect on all milk production traits decreasing 305-day milk, fat and protein yields by -8.85 kg, -0.53 kg and -0.33 kg, respectively. Classical, ancestral and new inbreeding generally had a detrimental effect on fertility and survival traits. From this study, it appears that Irish Holstein-Friesians have purged some of their genetic load for milk production through many years of selection based on production alone, while fertility, which has been less intensely selected for in the population demonstrates no evidence of purging.

## Introduction

Inbreeding is defined as the probability that two alleles at any locus are 'identical by descent' [1] and occurs when related individuals are mated. Inbreeding results in an increase in the number of homozygous loci [2], which may lead to an increase in the accumulation of recessive alleles. Mendelian factors unfavourable to fitness are more frequently recessive than dominant for two reasons: firstly, mutations tend to have negative effects on fitness and secondly, because dominant mutations will be quickly selected out of populations. This will lead to an accumulation of deleterious recessive alleles [2]. The loss in performance and vitality associated with inbreeding is termed "inbreeding depression" [3] and has generally been shown to be unfavourable [4].

It has been considered for different traits that dominance [3], overdominance [5], and epistatic effects [6] influences inbreeding depression. Where the genes are governed by dominance, inbreeding depression is caused by an increase in the number of genes with homozygous deleterious recessive genotypes and the decline in performance is expected to be linear with respect to the inbreeding coefficient [6]. Where traits are governed by overdominance, heterozygotes perform better than either homozygote, thus as inbreeding increases the number of homozygous loci, the proportion of advantageous heterozygous loci decreases. Epistasis occurs when an allele at one locus has an effect on an allele at another locus. Where epistatic interactions exist, a non-linear effect on performance is expected with respect to inbreeding [7]. The non-linear interactions are explained by the interaction deviation of double or multiple heterozygotes [8].

Regardless of the mechanisms underlying inbreeding depression, the effects of inbreeding are not consistent across populations or even sub-populations. Thus the level of inbreeding depression experienced is dependent, amongst other factors, on the genetic load of individuals [9].

Purging of inbreeding depression is a process whereby inbred animals with good performance have been selected from the population as parents, while the poorly performing inbred animals have not been selected. This biases the regression of inbreeding on performance and therefore estimates of inbreeding depression may be regressed towards zero [10]. Purging has been used as a tool in the breeding programme of the captive Speke's Gazelle to reduce the inbreeding depression expressed [11]; however it is also thought that purging occurs non-deliberately both in the natural world and in populations subject to selective pressures. Purging has been noted in several captive populations [12,10] as well as a feral herd of cattle [13] and has also been discussed using simulated populations [14-16]. Studies, which have investigated the efficacy of artificial selection to purge genetic load, found that after an initial positive response to selection there was a reverse response to selection [17,18]. However the authors could find no studies that attempted to quantify if purging existed in natural populations that have been subjected to stringent selective pressures such as in dairy cattle populations.

This study aims to fill this gap in knowledge. Evidence of purging in a population under performance-related selection such as the Irish population of Holstein-Friesians may help to explain the varying degrees of inbreeding depression observed in different livestock sub-populations globally, in addition to being of a subject of interest from a conservation genetics perspective.

## Methods

### Data edits

Pedigree information on 3,581,380 Holstein-Friesian animals as defined previously [19] was extracted from the Irish Cattle Breeding Federation database. In addition, all available milk production and fertility performance records for these animals were collated.

Lactation information from animals of parities 1 to 5 was retained and milk, fat and protein 305-d yields were predicted using standard lactation curves methodology as outlined by Olori *et al*. [20]. Lactations shorter than 100 d or longer than 400 d were removed. Outliers for milk production were defined as those greater than three standard deviations from the mean and were subsequently removed. Age was centred within parity and outlying ages deviating greater than 24 months from the median age per parity were removed as were animals younger than 20 months at first calving. Age at first calving, calving interval from first to second lactation and survival to second lactation were also calculated. In Ireland, the majority of dairy herds calve cows in the spring, although a small percentage operate a split calving pattern with a proportion of cows calving in the spring and the remainder calving in the autumn. To avoid potential bias on model solutions due to farmers consciously allowing an extended voluntary waiting period post-calving prior to insemination, only herd-years with at least 80% of animals calving between December and June inclusive were retained. Age at first calving was retained where animals calved between 660 and 900 days of age. Only calving intervals to second parity between 300 and 800 days were retained. Survival to second lactation was treated as a binary variable where animals who did not survive to second lactation were assumed to be culled (*i.e*. survival = 0).

For the analysis of milk production, calving interval and survival, contemporary groups of herd-year of calving were generated by concatenating herd and year of calving. For the analysis of age at first calving, contemporary groups of herd-year of birth were generated. Any contemporary group with less than five records was removed. Contemporary groups were generated to reduce the environmental variation caused by different management practises across different herd years.

Only animals with at least three complete generations of pedigree information were retained. To investigate the occurrence of purging, a deeper pedigree may be beneficial. However restriction of the data in the present study to animals with at least five complete generations of pedigree resulted in very small datasets and therefore analyses were restricted to animals with at least three complete generations of pedigree.

The remaining 88,366 records in the milk production data set had a mean 305-day milk, fat and protein yield of 6,878 kg (SD = 1,445.2 kg), 256 kg (SD = 55.0 kg) and 228 kg (SD = 45.4 kg), respectively. The fertility data set included 35,013 records on calving interval, 33,060 records on age at first calving and 39,741 records on survival to second lactation. These records had a mean calving interval, age at first calving and survival of 411 days (SD = 89.5 days), 763 days (SD = 52.9 days) and 0.78 respectively.

### Coefficients

Classical inbreeding coefficients were computed and ancestral inbreeding coefficients were computed using the algorithms of Ballou [12] and Kalinowski *et al*. [21], for all animals with production or fertility records available. All inbreeding coefficients were computed using the GRAIN programme from the software package Pedig [22], which computes inbreeding coefficients by simulation and gene dropping. In the present study 10,000 simulations were used and correlations between all inbreeding coefficients were tested.

Ballou [12] defines ancestral inbreeding as follows:

$$F_{a}=\frac{{\text{[F}}_{\text{a(s)}}\;+{\text{ (1-F}}_{\text{a(s)}}{\text{) F}}_{\text{s}}\;+{\text{ F}}_{\text{a(d)}}\;+{\text{ (1-F}}_{\text{a(d)}}{\text{)F}}_{\text{d}}\text{]}}{2}$$

where *F *and *F*_*a *_are the inbreeding coefficient and ancestral inbreeding coefficient, respectively, and the subscripts *s *and *d *represent the individuals sire and dam, respectively. The ancestral inbreeding coefficient as defined by Ballou [12] is the cumulative proportion of an individual's genome that has been previously exposed to inbreeding in its ancestors. Thus ancestral inbreeding arising from all common ancestors throughout the individual's pedigree is included in its Ballou ancestral inbreeding coefficient regardless of their contribution to the classical inbreeding coefficient. The correlation between Ballou ancestral inbreeding and classical inbreeding was relatively weak across both data sets and ranged from 0.36 to 0.40.

**Figure 1:**
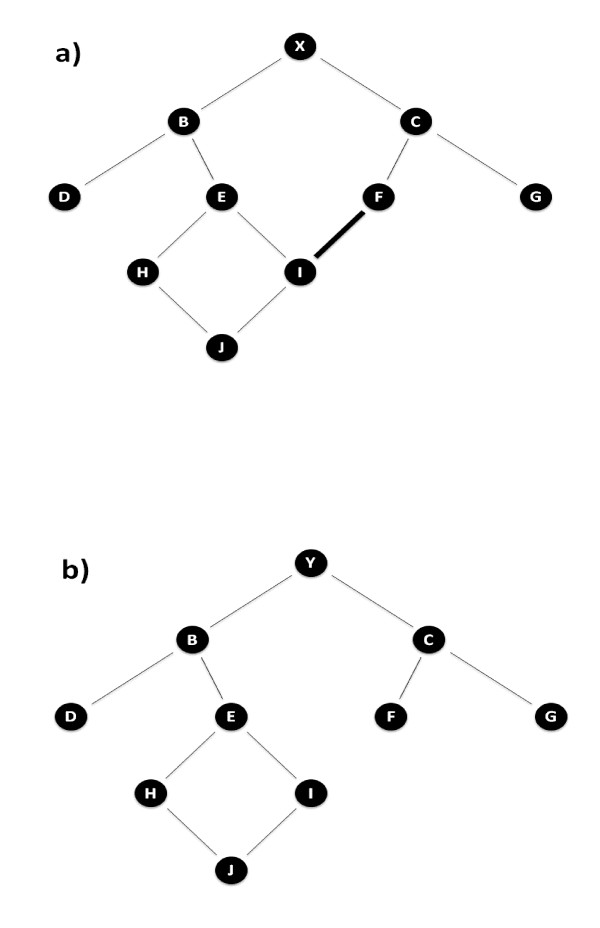
**Computation of classical, new and ancestral inbreeding coefficients for two individuals with similar pedigrees**. The difference between the pedigree of X and Y is the relationship between individual F and I. In Figure 1a, X has a classical inbreeding = 3.90%, new inbreeding = 3.51%, Ballou ancestral inbreeding = 3.13% and Kalinowski ancestral inbreeding = 0.39%. In Figure 1b, Y has a classical inbreeding = 0%, new inbreeding = 0%, Ballou ancestral inbreeding = 3.13% and Kalinowski ancestral inbreeding = 0%.

The algorithm of Kalinowski *et al*. [21] divides the classical inbreeding coefficient into two components, that where alleles are homozygous as they have met in the past (ancestral inbreeding), and that where alleles have met for the first time (new inbreeding). The Kalinowski ancestral inbreeding coefficient only includes the ancestral inbreeding of relationships whereby the common ancestor is on both sides of the pedigree (*i.e*. sire line and dam line). Hence when the classical inbreeding coefficient of an animal is 0, the Kalinowski ancestral inbreeding coefficient is also 0 (Fig. 1). The correlation between Kalinowski ancestral inbreeding and classical inbreeding was strong at 0.99 across both data sets and was to be expected because of the part-whole relationship between them.

The correlation between Ballou ancestral inbreeding and Kalinowski ancestral inbreeding was weak ranging from 0.28 to 0.38 across both data sets indicating that the two coefficients are measuring different population statistics. Figure 1 illustrates the difference between Ballou ancestral inbreeding and Kalinowski ancestral inbreeding.

"New" inbreeding coefficients were also computed for all animals according to Kalinowski *et al*. [21]. New inbreeding refers to that part of the classical inbreeding coefficient, which remains when the portion of the classical inbreeding coefficient explained by ancestral inbreeding has been removed. Therefore new inbreeding is the part of the classical inbreeding coefficient whereby alleles are homozygous and identical by descent but have not met already in the pedigree. Similar to the Kalinowski ancestral inbreeding coefficient, when the classical inbreeding coefficient is zero, the new inbreeding coefficient is also zero and strong correlations between new inbreeding and classical inbreeding of 0.81 and 0.82 were observed across data sets.

The ancestral inbreeding coefficient according to Ballou [12] will hereafter be referred to as Ballou, the ancestral inbreeding coefficient according to Kalinowski *et al*. [21] will hereafter be referred to as Kalinowski, and that part of the classical inbreeding coefficient denoting new inbreeding, as defined by Kalinowski *et al*. [21] will hereafter be referred to as new inbreeding.

### Analysis

All analyses were undertaken in ASReml [23]. The sire models used to estimate inbreeding depression and purging were:

Y1_ijklmn _= μ + HY_i _+ MTH_j _+ PAR_k _+ AGE_l_(PAR_k_) + INB_m _+ S_n _+ e_ijklmn_;

Y2_ijmn _= μ + HY_i _+ MTH_j _+ INB_m _+ S_n _+ e_ijmn_;

where Y1_ijklmn _is lactation milk, fat, and protein yield (kg), Y2_ijmn _is calving interval (days), age at first calving (days) or survival (0/1), μ is the mean of the population, HY_i _is the fixed effect of herd-year of calving/birth *i*, MTH_j _is the effect of month of calving/birth *j*, PAR_k _is the dam parity (*k *= 1 to 5), AGE_l _(PAR_k_) is the age in months *l *centered within parity *k*, INB_m _is one of either the classical, ancestral, or new inbreeding coefficient of animal *m*, S_n _is the random effect of sire *n *and e is the random residual effect. A favourable ancestral inbreeding regression coefficient significantly different from zero suggests the occurrence of purging of inbreeding depression for the trait under investigation [12], while an unfavourable classical or new inbreeding regression coefficient significantly different from zero indicates inbreeding depression of the trait.

In addition to the models described above, two variations were also tested. Firstly, the classical inbreeding coefficient was included in the model together with either the Ballou ancestral inbreeding coefficient or the new inbreeding coefficient. Colinearity existed between Kalinowski ancestral inbreeding and classical inbreeding as estimated using the condition index in PROC REG (SAS^® ^Inst. Inc. Cary, NC). Thus Kalinowski ancestral inbreeding was not included as a covariate in the model when classical inbreeding was also included. This model, including the classical inbreeding coefficient and either the Ballou ancestral inbreeding coefficient or the new inbreeding coefficient, simultaneously, was solved to detect any changes in the regression coefficient of milk production and fertility on classical inbreeding when ancestral inbreeding or new inbreeding was also included in the model. If classical inbreeding has a greater detrimental effect on performance following the removal of the ancestral inbreeding effect, it may suggest purging of inbreeding depression, as it indicates that ancestral inbreeding has a positive effect on performance and weakens the detrimental effect of classical inbreeding on performance. If classical inbreeding has less of a detrimental effect on performance following the removal of the new inbreeding effect, it may also suggest purging of inbreeding depression as it indicates that new inbreeding enhances the detrimental effect of classical inbreeding.

The second model variation tested the interaction between Ballou ancestral inbreeding and classical inbreeding. In this model, classical inbreeding was included as a continuous variable in itself, while ancestral inbreeding was only included in a two-way interaction with classical inbreeding. This is similar to the model described by Ballou [12] whereby the performance of non-inbred animals is independent of the ancestral inbreeding coefficient, yet ancestral inbreeding can affect the inbreeding effect. Favourable coefficients for the interaction suggest the occurrence of purging of inbreeding depression for that trait [12].

Comparisons between each of the regression coefficients obtained for the three model variations, together with their respective standard errors were used to determine the differences between the models.

## Results

The majority of animals used in the analyses were inbred with 97.0% and 97.6% of animals inbred in the milk production and fertility data sets, respectively. The average complete generation equivalent of the animals was 6.29 for the milk production data set and 6.46 for the fertility data set. Mean classical, new and ancestral inbreeding coefficients for all animals in each of the data sets are presented in Table 1. Average new inbreeding (0.43 to 0.46%) was considerably lower than average classical inbreeding (2.58 to 2.68%) across all data sets indicating that the majority of the average classical inbreeding coefficient is explained by alleles homozygous and identical by descent from a past meeting in an animal's pedigree. As the coefficient of the Ballou measure of ancestral inbreeding [12] includes all common ancestors in the pedigree of an individual, regardless of their contribution to the classical inbreeding coefficient, average Ballou ancestral inbreeding coefficients (6.50 to 6.89%) were considerably larger than average Kalinowski ancestral inbreeding coefficients (2.15 to 2.22%) across data sets (Table 1).

**Table 1:** Mean coefficients (%) of new inbreeding (New) and ancestral inbreeding as defined by Kalinowski *et al*. [21] and Ballou [12] across all data sets analysed^1^

	New	Kalinowski	Ballou
Milk production	0.43	2.15	6.50
Calving interval	0.46	2.22	6.85
Age at first calving	0.46	2.21	6.89
Survival	0.46	2.22	6.84

^1^Classical inbreeding = New inbreeding + Kalinowski ancestral inbreeding

### Inbreeding effects on milk production

Table 2 summarises the regression coefficients of classical inbreeding, new inbreeding, Kalinowski and Ballou ancestral inbreeding and the interaction between Ballou ancestral inbreeding and classical inbreeding on milk production performance. Table 3 summarises how including either Ballou ancestral or new inbreeding in the model affected the regression coefficient on classical inbreeding.

**Table 2:** The effect of classical inbreeding (*F*), new inbreeding (New), ancestral inbreeding as defined by Kalinowski *et al*. [21] and Ballou [12.] As well as the interaction between classical inbreeding and Ballou ancestral inbreeding (F*Ballou) on milk, fat and protein yield (kg) for all data sets when each of these terms were individually included in a multiple regression model with confounding effects adjusted for^1,2^.

	*F*	New	Kalinowski	Ballou	F*Ballou^3^
Milk	**-7.63 (1.442)**	**-32.41 (8.328)**	**-8.85 (1.662)**	**4.85 (1.602)**	0.34 (0.309)
Fat	**-0.47 (0.057)**	**-2.43 (0.328)**	**-0.53 (0.066)**	-0.06 (0.063)	-0.01 (0.012)
Protein	**-0.28 (0.045)**	**-1.11 (0.259)**	**-0.33 (0.052)**	**0.12 (0.050)**	0.02 (0.010)

^1^Regression coefficients in bold type are significantly (*P *< 0.05) different from zero

^2 ^S.E. in brackets

^3 ^Regression coefficient for the interaction of classical inbreeding*Ballou ancestral inbreeding following adjustment for classical inbreeding

**Table 3:** The regression coefficient of classical inbreeding (*F*) when included in the multiple regression model alone, as well as the regression coefficients of classical and new inbreeding, and classical and ancestral inbreeding as defined by Ballou [12] when included in the model simultaneously, on milk, fat and protein yield (kg) ^1,2^

	*F*	New^3^		Ballou^4^	
		
		New	*F*	Ballou	*F*
Milk	**-7.51 (1.472)**	5.63 (14.170)	**-8.29 (2.454)**	**6.6 (1.678)**	**-8.86 (1.511)**
Fat	**-0.47 (0.058)**	-0.75 (0.555)	**-0.37 (0.096)**	0.04 (0.066)	**-0.48 (0.059)**
Protein	**-0.27 (0.046)**	0.42 (0.439)	**-0.33 (0.076)**	**0.18 (0.052)**	**-0.31 (0.047)**

^1^Regression coefficients in bold type are significantly (*P *< 0.05) different from zero

^2 ^S.E. in brackets

^3 ^Regression coefficients for new inbreeding and classical inbreeding when included simultaneously in the model

^2 ^Regression coefficients for ancestral inbreeding as defined by Ballou [12] and classical inbreeding when included simultaneously in the model.

Ballou ancestral inbreeding had a positive effect on milk and protein yield, while the interaction between Ballou ancestral inbreeding and classical inbreeding showed similar trends although results were not significantly different from zero (Table 2). Classical inbreeding had a numerically greater detrimental effect on milk, fat and protein yield when the Ballou ancestral inbreeding term was also included in the model (Table 3). However results were generally not significantly different to when only classical inbreeding was included in the model.

Kalinowski ancestral inbreeding had an unfavourable effect on milk production (Table 2). Regressions of milk production on Kalinowski ancestral inbreeding were all negative and greater than, although not significantly different from, the regression of milk production performance on classical inbreeding. As new inbreeding increased, there was a strong detrimental effect on milk production and the effect was greater than that observed for the effect of classical inbreeding (Table 2).

### Inbreeding effects on fertility

The effect of the different definitions of inbreeding on calving interval, age at first calving and survival are presented in Table 4. Increases in new inbreeding were associated with a much greater increase in calving interval (*P *< 0.05) and age at first calving (*P *< 0.001) than the increases associated with the classical inbreeding coefficient. Both Kalinowski and Ballou ancestral inbreeding had an unfavourable effect on calving interval, age at first calving and survival and were similar in magnitude to the effect of classical inbreeding on the respective traits (Table 4). The interaction between Ballou ancestral inbreeding and classical inbreeding showed a similar trend to the effect of Ballou ancestral inbreeding when included as a separate effect. There was little change in the effect of classical inbreeding on fertility and survival when either the new or ancestral inbreeding term was included in the model (Table 5).

**Table 4:** The effect of classical inbreeding (*F*), new inbreeding (New), ancestral inbreeding as defined by Kalinowski *et al*. [21] and Ballou [12], as well as the interaction between classical inbreeding and Ballou ancestral inbreeding (F*Ballou) on calving interval (days), age at first calving (days) and survival to second lactation (0/1*100) when each of these terms were individually included in a multiple regression model with confounding effects adjusted for^1,2^.

	*F*	New	Kalinowski	Ballou	F*Ballou^3^
Calving interval	**0.54 (0.220)**	**4.14 (1.253)**	**0.55 (0.254)**	**0.81 (0.213)**	**0.09 (0.044)**
Age at first calving	**0.48 (0.096)**	**3.09 (0.582)**	**0.52 (0.110)**	**0.44 (0.105)**	**0.05 (0.021)**
Survival	**-0.29 (0.092)**	-1.03 (0.533)	**-0.35 (0.106)**	-0.16 (0.090)	0.01 (0.019)

^1^Regression coefficients in bold type are significantly (*P *< 0.05) different from zero

^2 ^S.E. in brackets

^3 ^Regression coefficient for the interaction of classical inbreeding*Ballou ancestral inbreeding following adjustment for classical inbreeding.

**Table 5:** The regression coefficient of classical inbreeding (*F*) when included in the multiple regression model alone, as well as the regression coefficients of classical and new inbreeding, and classical and ancestral inbreeding as defined by Ballou [12] when included in the model simultaneously, on calving interval (days), age at first calving (days) and survival to second lactation (0/1*100)^1,2^

	*F*	New^3^		Ballou^4^	
		New	*F*	Ballou	*F*
Calving Interval	**0.54 (0.220)**	**4.76 (2.123)**	-0.13 (0.372)	**0.72 (0.220)**	0.38 (0.225)
Age at first calving	**0.48 (0.096)**	**2.09 (0.989)**	0.20 (0.163)	**0.33 (0.108)**	**0.43 (0.098)**
Survival	**-0.29 (0.092)**	0.93 (0.885)	**-0.42 (0.153)**	-0.09 (0.093)	**-0.27 (0.095)**

^1^Regression coefficients in bold type are significantly (P < 0.05) different from zero

^2 ^S.E. in brackets

^3 ^Regression coefficients for new inbreeding and classical inbreeding when included simultaneously in the model

^4^Regression coefficients for ancestral inbreeding as defined by Ballou [12] and classical inbreeding when included simultaneously in the model.

## Discussion

Classical inbreeding, new inbreeding, and two definitions of ancestral inbreeding as well as the interaction between ancestral inbreeding and classical inbreeding were compared for their effect on milk production, fertility and survival performance in Irish Holstein-Friesian dairy cows. It was postulated that the Irish Holstein-Friesian population would be likely to undergo purging as populations which experience a slow rate of increase in inbreeding over time are more likely to show the effects of purging [24] and the rate of increase of inbreeding in the Irish Holstein-Friesian population is relatively low at 0.1% per annum [19]. Furthermore, the Holstein-Friesian population has undergone intense selection over past decades. Thus the aim of this study was to determine if purging existed within the Irish Holstein-Friesian population.

### Inbreeding effects on milk production

Evidence of purging, as defined by improved performance associated with increased ancestral inbreeding is evident in the Irish population of Holstein-Friesians for milk production. However, results are not consistent across all milk production traits or definition of ancestral inbreeding coefficient. The greater effect of new inbreeding on milk production compared to classical inbreeding (Table 2) indicates that the most detrimental portion of the classical inbreeding coefficient is that portion attributable to new inbreeding. This is further substantiated by the positive effect of Ballou ancestral inbreeding on milk and protein yield (Table 2) and indicates purging [10]. The favourable effect of ancestral inbreeding suggests that the increased homozygosity caused by increased inbreeding may be at loci which have a minor or no unfavourable effect on milk production. Results from this study show that inbreeding depression, as defined by classical inbreeding, was greater when Ballou ancestral inbreeding was accounted for in the model (Table 3), despite the ancestral inbreeding not always having a positive effect on performance in itself (Table 2).

In contrast to the regression of milk production on new inbreeding and Ballou ancestral inbreeding, Kalinowski ancestral inbreeding had an unfavourable effect on milk production. As the Ballou ancestral inbreeding coefficient details all common ancestors in the pedigree of individuals and not just those common ancestors crossing over between the sire and dam lines as in the Kalinowski ancestral inbreeding coefficient, it is possible that the Ballou ancestral inbreeding coefficient may provide a better representation of the evidence of purging.

### Inbreeding effects on fertility

The evidence of purging within the fertility data sets is less convincing. New inbreeding was associated with a significantly greater increase in calving interval and age at first calving than the increase associated with classical inbreeding (Table 4). However, both Kalinowski and Ballou ancestral inbreeding were also associated with unfavourable effects on fertility and survival performance (Table 4). This is consistent with the selection history for the ancestors of these animals.

Since 2001 in Ireland, genetic merit of dairy animals has been quantified using a total merit index, namely the Economic Breeding Index (EBI), which comprises of five sub-indices: milk production, fertility, calving performance, health and beef production [25]. Prior to 2001, selection was primarily based on a production index and a large amount of Holstein-Friesian germplasm was imported into Ireland during this period from countries where the main breeding goal was production [26]. This contributed to the purging effect in milk production since stochastic simulations undertaken by Hedrick [27] showed that purging was most successful when high selection pressure was imposed. As an antagonistic relationship exists between milk production and fertility [28], the fertility of these animals may have been genetically compromised. Therefore the ancestral inbreeding coefficient associated with these inbred ancestors is likely to have a detrimental effect on fertility. Additionally any increases in homozygosity from greater inbreeding in selected animals of past generations may have had no effect on production but could have had a deleterious effect on fertility, which went unnoticed. Another possible reason for the lack of a favourable effect of ancestral inbreeding on fertility may be due to inbreeding depression in fertility being predominantly due to overdominance or associative overdominance [12].

Mc Parland *et al*. [4] previously investigated the effect of inbreeding on several milk production, calving performance, conformation and fertility traits. They found that the greatest effect of inbreeding was for fertility. The results from this study offer an explanation for that finding. In contrast to milk production, where a positive effect of Ballou ancestral inbreeding on milk production was evident (Table 2), beneficial effects of ancestral inbreeding on fertility have not yet become established in the pedigree of the current Irish Holstein-Friesian population due to the lack of selection on fertility. Therefore the entire inbreeding coefficient has a negative effect on fertility performance, not just that part of the inbreeding coefficient explained by new inbreeding.

## Conclusion

This study demonstrates that purging is likely to have occurred in the Irish Holstein-Friesian population for milk production, but not for fertility. This is consistent with the history of selection in the Irish Holstein-Friesian population, whereby only the more recent generations have been selected for improved fertility. For a thorough analysis of effect of purging on milk production and fertility of cattle, a large population with a deep well-recorded pedigree is an essential requirement.

## Competing interests

The authors declare that they have no competing interests.

## Authors' contributions

SMP conceived the original idea of the study, undertook all the statistical analyses and wrote the manuscript. FK helped with the statistical analyses and undertook a through review of the manuscript. DPB further developed the original idea, helped in the data handling, editing and analysis as well as the writing of the manuscript. All authors approved the final version.
